# Hypoxia-induced OPN5 expression is associated with matrix-preserving responses in human scleral fibroblasts

**DOI:** 10.3389/fnins.2026.1896819

**Published:** 2026-08-10

**Authors:** Maolan Tang, Sha Lu, Wenjun Zhou, Jianwei Li, Hua Li

**Affiliations:** 1 Department of Ophthalmology, Yongchuan Hospital of Chongqing Medical University (The fifth Clinical College of Chongqing Medical University), Chongqing, China; 2 Yongchuan District Key Laboratory of Optometry, Chongqing, China; 3 General Practice School of Chongqing Medical University, Chongqing, China; 4 Central Laboratory, Yongchuan Hospital of Chongqing Medical University (The fifth Clinical College of Chongqing Medical University), Chongqing, China; 5 The Geriatric Diseases Clinical Medical Research Center of Chongqing, Yongchuan Hospital of Chongqing Medical University (The fifth Clinical College of Chongqing Medical University), Chongqing, China

**Keywords:** hypoxia, myopia, Opn5, scleral remodeling, violet light

## Abstract

**Introduction:**

Myopia-associated scleral remodeling is characterized by altered extracellular matrix (ECM) turnover and progressive biomechanical weakening, processes that have been increasingly linked to scleral hypoxia. Opsin 5 (OPN5), a violet light-sensitive opsin, has been implicated in ocular growth regulation, but its role in scleral fibroblasts remains largely unknown. This study investigated the expression of OPN5 in human scleral fibroblasts (HSFs) under hypoxic conditions and examined its association with remodeling-related cellular responses.

**Methods:**

Primary HSFs were cultured under hypoxia (2% O_2_) and exposed to violet light (360–400 nm, 400 μW/cm^2^). Expression of OPN5 and remodeling-associated markers, including COL1A1, MMP2, TIMP2, and α-SMA, was assessed by quantitative real-time PCR and Western blotting. OPN5 gain- and loss-of-function models were generated to evaluate associations between OPN5 expression and ECM-related responses. Transcriptomic profiling was performed in OPN5-overexpressing cells to explore potentially associated biological pathways.

**Results:**

Exposure to hypoxic conditions resulted in progressive upregulation of OPN5 at both the transcript and protein levels and was accompanied by reduced COL1A1 and TIMP2 expression together with increased MMP2 and α-SMA expression, consistent with a remodeling-associated phenotype. Violet light further enhanced OPN5 expression under hypoxic conditions and was associated with partial restoration of COL1A1 and TIMP2 expression, together with reduced MMP2 and α-SMA expression levels. OPN5 overexpression was associated with increased COL1A1 and TIMP2 expression and decreased MMP2 and α-SMA expression, whereas CRISPR/Cas9-mediated reduction of endogenous OPN5 was associated with decreased COL1A1 and TIMP2 together with increased α-SMA expression. Transcriptomic analysis suggested potential associations between OPN5 expression and pathways related to extracellular matrix regulation, metabolism, Wnt signaling, and cytokine responses.

**Conclusion:**

OPN5 expression was increased in human scleral fibroblasts under hypoxic conditions and was associated with molecular signatures consistent with extracellular matrix preservation and reduced fibroblast activation. Violet light further enhanced OPN5 expression and was associated with partial attenuation of hypoxia-associated remodeling changes. These findings suggest that OPN5 is a hypoxia-responsive factor in scleral fibroblasts and support a context-dependent association between OPN5 signaling and extracellular matrix homeostasis under conditions relevant to myopia-associated scleral remodeling.

## Introduction

Myopia is one of the most prevalent ocular disorders worldwide and a leading cause of irreversible visual impairment, particularly in its high forms, which are associated with complications such as retinal detachment, glaucoma, and myopic maculopathy (Dolgin, 2015; Du et al., 2021; Foo et al., 2023; Haarman et al., 2020; Holden et al., 2016; Jonas et al., 2024; Morino et al., 2026). Progressive axial elongation is considered the structural basis underlying myopia development, and increasing evidence suggests that the sclera plays a central role in regulating ocular size through dynamic extracellular matrix (ECM) remodeling (Boote et al., 2020; Lin et al., 2024a; McBrien et al., 2009; Wu et al., 2018; Zhao et al., 2020).

Myopia-associated scleral remodeling is characterized by scleral thinning, reduced biomechanical strength, and altered ECM organization (Boote et al., 2020; Huang et al., 2019; McBrien et al., 2009; Ouyang et al., 2019). At the molecular level, these changes are closely associated with dysregulated collagen turnover. Type I collagen, primarily encoded by COL1A1, is the major structural component of the sclera and is essential for maintaining tissue integrity (Gentle et al., 2003; Li et al., 2024; Ouyang et al., 2019; Zhu H. et al., 2025). Experimental myopia is commonly accompanied by reduced collagen synthesis together with increased matrix degradation (Ikeda et al., 2022; Li et al., 2025; Lin et al., 2024b; Wen et al., 2025; Zhu H. et al., 2025). Matrix metalloproteinase-2 (MMP2), a key mediator of ECM degradation, is frequently upregulated during myopia progression, whereas tissue inhibitor of metalloproteinase-2 (TIMP2) functions to restrain MMP2 activity (Hui et al., 2026; Li et al., 2025; Liu et al., 2017, 2025). In parallel, increased α-smooth muscle actin (α-SMA) expression reflects fibroblast activation and acquisition of a more contractile cellular phenotype (Jobling et al., 2009; Qiu et al., 2018; Yuan et al., 2018). Together, these molecular changes are thought to contribute to weakening of the scleral matrix and reduced resistance to axial elongation.

Among the factors implicated in scleral remodeling, hypoxia has attracted increasing attention (Feng et al., 2025; Meng et al., 2025; Wu et al., 2018, 2022; Xiao et al., 2025; Zhang et al., 2026; Zhao et al., 2020, 2025). Choroidal thinning during myopia progression may reduce oxygen delivery to the posterior sclera, thereby exposing scleral fibroblasts to a relatively hypoxic microenvironment (Wu et al., 2018; Xiao et al., 2025; Zhang et al., 2025; Zhao et al., 2020). Consistent with this concept, activation of hypoxia-associated signaling pathways, including HIF-related mechanisms, has been linked to enhanced ECM remodeling and altered fibroblast behavior (Wu et al., 2022; Zhao et al., 2020, 2025). However, the molecular pathways through which scleral fibroblasts integrate hypoxic stress with environmental signals relevant to eye growth regulation remain incompletely understood.

Light exposure is one of the most reproducible environmental factors associated with reduced myopia progression (Cohen et al., 2011; He et al., 2015, 2022; Rose et al., 2008; Shi et al., 2025). In particular, violet light has been reported to suppress experimental myopia in animal models and may influence ocular growth regulation (Jiang et al., 2021; Strickland et al., 2020). Opsin 5 (OPN5, neuropsin) is a violet light-sensitive opsin that couples to Gi-type G proteins (Kojima et al., 2011; Yamashita et al., 2010, 2025) and is expressed in multiple ocular tissues (Buhr et al., 2015; Díaz et al., 2020; Kato et al., 2019). Recent studies have implicated OPN5 in light-responsive signaling pathways involved in ocular development and circadian regulation (Buhr et al., 2015; Jiang et al., 2021; Nguyen et al., 2019). In animal models, retinal OPN5-expressing cells have been associated with violet light-dependent suppression of experimental myopia (Jiang et al., 2021; Strickland et al., 2020). In addition, OPN5 has been localized in the embryonic chick eye, where it influences the expression of multiple myopia-related genes, suggesting a conserved role of OPN5 in ocular growth regulation across species (Kato et al., 2019).

Although most studies have focused on retinal mechanisms, scleral fibroblasts represent the ultimate effector cells responsible for extracellular matrix remodeling during axial elongation. Nevertheless, most current studies have focused primarily on retinal or neural mechanisms, whereas the potential involvement of OPN5 in scleral remodeling-associated pathways remains unclear.

In particular, whether OPN5 participates in hypoxia-associated responses in scleral fibroblasts has not been investigated. It also remains unclear whether violet light influences extracellular matrix-related pathways in scleral fibroblasts under hypoxic stress conditions. Addressing these questions may improve understanding of how environmental light-responsive pathways interact with local stress-associated remodeling processes in the sclera.

Here, we investigated the expression and potential involvement of OPN5 in human scleral fibroblasts (HSFs) under hypoxic conditions and examined the effects of violet light stimulation. We observed that hypoxia induced both OPN5 expression and remodeling-associated molecular changes in HSFs. Violet light further enhanced OPN5 expression and was associated with partial reversal of several hypoxia-associated remodeling markers. In addition, modulation of OPN5 expression was associated with alterations in extracellular matrix-related markers. Together, these findings suggest a context-dependent association between OPN5 signaling and extracellular matrix homeostasis in scleral fibroblasts under hypoxic stress conditions relevant to myopia-associated remodeling.

## Methods

### Human scleral fibroblast culture and treatment

#### Cell culture

Primary human scleral fibroblasts (HSFs) and complete culture medium (Cat. No. SNPM-H089) were purchased from Wuhan Sunncell Biotechnology Co., Ltd. Cells were maintained according to the manufacturer’s instructions at 37 °C in a humidified incubator containing 5% CO_2_ under normoxic conditions unless otherwise specified. Cells from similar passage ranges (passage 2∼3) were used across experiments to minimize passage-dependent variability.

### Hypoxic treatment

To establish an in vitro hypoxia-associated remodeling model, HSFs were cultured in a tri-gas incubator under 2% O_2_, 5% CO_2_, and balanced N_2_ at 37 °C for 12, 24, 48, or 72 h, as indicated in each experiment. Cells maintained under normoxic culture conditions served as controls. At the indicated time points, cells were collected for protein or RNA analyses.

### Violet light irradiation

For violet light stimulation experiments, HSFs cultured under hypoxic or normoxic conditions were exposed to violet light (360–400 nm; irradiance: 400 μW/cm^2^) for 3 h per irradiation session directly inside the incubator, ensuring that the designated oxygen tension was maintained throughout the irradiation period. Irradiance was measured using a calibrated radiometer before each experiment. Cells received violet light exposure at 6 h, 54 h, or both 6 h and 54 h after initiation of hypoxic or normoxic culture. In hypoxia-associated experiments, the total hypoxic exposure duration was maintained at 72 h across all groups. Following irradiation, cells remained in the incubator until sample collection. Temperature was monitored throughout irradiation and no measurable increase was detected (Supplementary Figure 1).

### Detection of intracellular reactive oxygen species (ROS)

Intracellular ROS levels were measured using the Reactive Oxygen Species Assay Kit (S0033S, Beyotime) according to the manufacturer’s instructions. Briefly, following the indicated treatments, cells were washed once with pre-warmed phosphate-buffered saline (PBS) and incubated with 10 μM 2′, 7′-dichlorodihydrofluorescein diacetate (DCFH-DA) diluted in serum-free DMEM for 30 min at 37 °C in the dark. After incubation, cells were washed three times with PBS to remove excess probe. Intracellular ROS levels were evaluated by detecting DCF fluorescence using a fluorescence microscope (Ex/Em = 488/525 nm). Representative fluorescence images (Supplementary Figure 1C) were acquired under identical imaging settings for all experimental groups.

### Western blot analysis

Total cellular protein was extracted using lysis buffer containing 8 M urea, 2% CHAPS, and 20 mM dithiothreitol (DTT), supplemented with protease inhibitors (1 mM PMSF, 1 μg/mL aprotinin, 1 μg/mL leupeptin, and 1 μg/mL pepstatin A). Protein concentrations were determined using a bicinchoninic acid (BCA) assay kit according to the manufacturer’s instructions.

Equal amounts of protein were separated by SDS–PAGE and transferred onto polyvinylidene difluoride (PVDF). Membranes were blocked with 5% non-fat milk in TBST for 2 h at room temperature and incubated overnight at 4 °C with primary antibodies against OPN5, COL1A1, MMP2, TIMP2, α-SMA, DRD4, TGF-β1, Bax, Bcl-2, β-actin, and other indicated proteins (Supplementary Table 1). After washing with TBST, membranes were incubated with horseradish peroxidase-conjugated secondary antibodies for 2 h at room temperature.

Protein bands were visualized using enhanced chemiluminescence reagents and imaged using a ChemiDoc XRS+ imaging system (Bio-Rad Laboratories, Hercules, CA, USA). Band intensities were quantified using ImageJ software and normalized to β-actin. All experiments were independently repeated at least three times.

### RNA extraction and quantitative real-time PCR

Total RNA was extracted using TRIzol reagent according to the manufacturer’s instructions. RNA concentration and purity were evaluated using a NanoDrop 2000 spectrophotometer. One microgram of total RNA was reverse-transcribed into cDNA using a commercial reverse transcription kit.

Quantitative real-time PCR (qRT-PCR) was performed using SYBR Green Master Mix on a CFX96 Real-Time PCR System (Bio-Rad Laboratories). Relative gene expression was normalized to β-actin and calculated using the 2^–ΔΔ*Ct*^ method. Primer sequences are listed in Supplementary Table 2. Each experiment included at least three independent biological replicates.

### Construction of OPN5-reduced cells

Single-guide RNA (sgRNA) targeting the human OPN5 gene was designed using the Benchling platform. The sgRNA sequence with the highest predicted targeting efficiency (5′-CAAAAGGATCCCCATCTCGA-3′) was cloned into the PX459 vector.

HSFs were transfected with the CRISPR/Cas9 plasmid using Lipofectamine 2000 according to the manufacturer’s protocol. At 24 h after transfection, cells were subjected to puromycin selection (1 μg/mL) for 24 h. Surviving cells were expanded for subsequent experiments. Although attempts were made to isolate monoclonal knockout cells by limiting dilution, stable monoclonal OPN5 knockout lines were not successfully established. Therefore, the resulting cell population with reduced OPN5 expression was used as a CRISPR-mediated OPN5-reduced model. Western blot densitometry demonstrated an approximately ∼71% reduction (Supplementary Figure 2) in OPN5 protein expression compared with control cells.

### Construction of OPN5-overexpressing cells

The full-length coding sequence of human OPN5 was amplified from HSF-derived cDNA and cloned into the pFBM expression vector using NheI and XhoI restriction sites. Recombinant plasmids were verified by Sanger sequencing.

HSFs were transfected with the OPN5 overexpression plasmid using Lipofectamine 2000, while cells transfected with the corresponding empty vector served as controls. After 6 h, the transfection medium was replaced with complete culture medium, and cells were maintained under standard culture conditions before downstream analyses.

### Transcriptomic analysis

Total RNA was extracted from control and OPN5-overexpressing HSFs using TRIzol reagent. RNA integrity and quality were evaluated using an Agilent 2100 Bioanalyzer. Samples meeting quality control criteria were subjected to library preparation and high-throughput RNA sequencing by Qiantang Biotechnology Co., Ltd.

After adaptor trimming and quality filtering, clean reads were aligned to the human reference genome (GRCh38/hg38) using HISAT2. Gene expression levels were quantified, and differential expression analysis was performed using DESeq2. Genes with |log_2_ fold change| ≥ 2 and adjusted *P* < 0.05 were considered differentially expressed. Heatmaps and enrichment analyses were generated using standard R packages including pheatmap and clusterProfiler.

### Statistical analysis

All experiments were performed using at least three independent biological replicates. Data are presented as mean ± SEM, as indicated in the figure legends. Statistical analyses were performed using GraphPad Prism version 10.1.2 (GraphPad Software, San Diego, CA, USA). Statistical analyses followed conventions commonly used in exploratory cell biology studies with independent biological replicates. For comparisons between two groups, unpaired two-tailed Student’s *t*-tests were used for normally distributed data. For comparisons involving more than two groups, one-way ANOVA followed by Tukey’s *post hoc* multiple-comparison test was applied. Non-parametric tests were used when normality assumptions were not met. *P* < 0.05 was considered statistically significant.

## Results

### Hypoxic conditions are associated with OPN5 upregulation and remodeling-associated changes in human scleral fibroblasts

**FIGURE 1:**
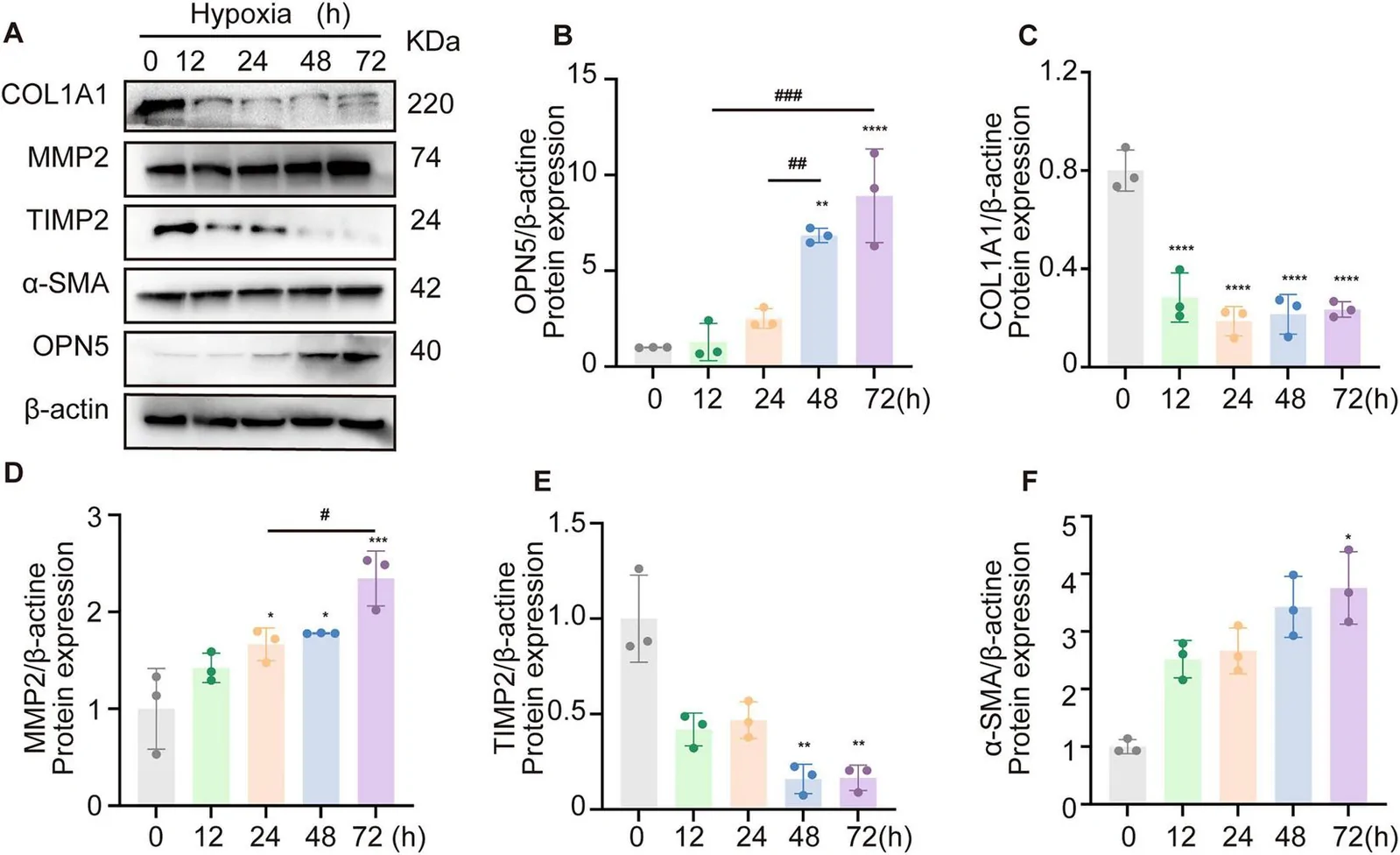
Hypoxia induces OPN5 expression and remodeling-associated molecular changes in human scleral fibroblasts. **(A)** HSFs were exposed to 2% O_2_, and the protein expression of COL1A1, MMP2, TIMP2, α−SMA and OPN5 was detected by Western blot, with β-actin serving as the internal loading control. **(B–F)** Quantitative bar graph analysis of relative protein expression of OPN5 **(B)**, COL1A1 **(C)**, MMP2 **(D)**, TIMP2 **(E)**, and α-SMA **(F)** (*n* = 3). Data are expressed as mean ± SEM. */^#^*P* < 0.05; **/^##^*P* < 0.01; ***/^###^*P* < 0.001; ****/^####^*P* < 0.001. Statistical differences were analyzed by one-way ANOVA followed by Tukey’s *post hoc* multiple comparison test. Treatment duration: 12, 24, 48, and 72 h compared with the control (0 h).

To investigate whether OPN5 responds to hypoxic stress in scleral fibroblasts, HSFs were cultured under hypoxic conditions for increasing durations. OPN5 protein expression progressively increased over time compared with normoxic controls (Figures 1A,B). Consistent changes were also observed at the transcript level (Supplementary Figure 3), indicating that OPN5 expression progressively increased under hypoxic conditions in HSFs.

Hypoxia was accompanied by coordinated alterations in extracellular matrix-related markers. COL1A1 protein expression decreased progressively under hypoxia (Figures 1A, C). At the transcript level, COL1A1 showed a transient increase at 24 h before declining at later time points (Supplementary Figure 3). In parallel, MMP2 expression increased whereas TIMP2 expression decreased under hypoxic conditions at both the protein and transcript levels (Figures 1A, D, E, Supplementary Figure 3D). α-SMA protein expression also increased during prolonged hypoxia (Figures 1A, F), consistent with molecular features of an activated fibroblast phenotype. Collectively, these findings suggest that progressive OPN5 upregulation occurs together with coordinated remodeling-associated molecular changes under hypoxic conditions in HSFs.

### Violet light is associated with increased OPN5 expression and partial reversal of hypoxia-related remodeling changes

**FIGURE 2:**
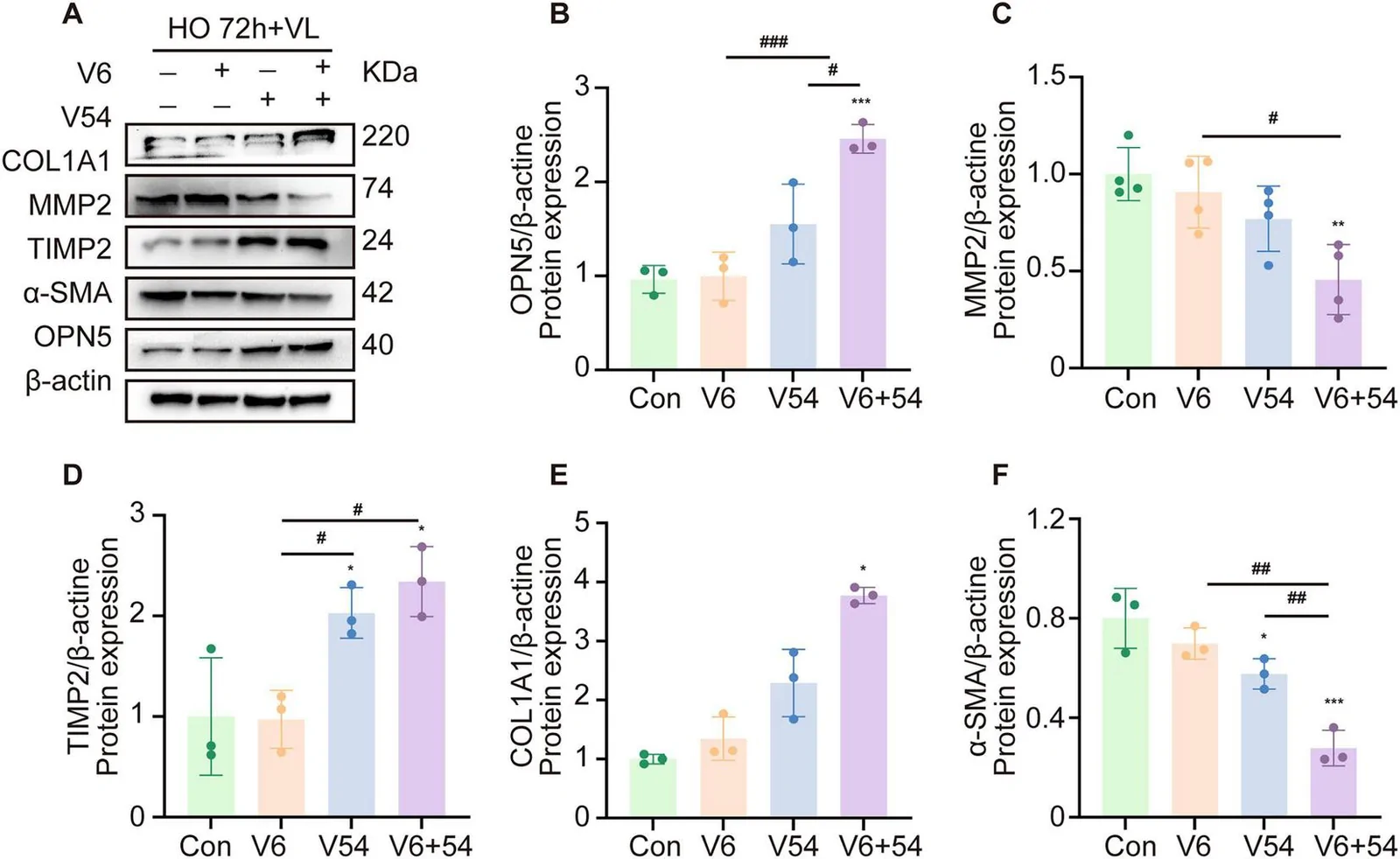
Effects of violet light irradiation on hypoxia-associated molecular changes in HSFs. **(A)** Representative Western blots of COL1A1, MMP2, TIMP2, α-SMA, OPN5, and β-actin in HSFs cultured under hypoxia (2% O_2_, 72 h) with or without violet light irradiation (400 μW/cm^2^, 3 h per session). Violet light was administered at 6 h (V6), at 54 h (V54), or at both time points (V6+V54). **(B–F)** Densitometric quantification of OPN5 **(B)**, MMP2 **(C)**, TIMP2 **(D)**, COL1A1 **(E)**, and α-SMA **(F)** protein expression relative to β-actin (*n* = 3). Violet light was associated with OPN5 expression and was associated with decreased MMP2 and α-SMA levels and increased TIMP2 and COL1A1 levels under hypoxic conditions, with the strongest effect observed in the dual-irradiation group. Data are shown as mean ± SEM. Statistical significance was determined by one-way ANOVA followed by Tukey’s *post hoc*-test. */^#^*P* < 0.05, **/^##^*P* < 0.01, and ***/^###^*P* < 0.001.

Because OPN5 is responsive to short-wavelength light, we next examined whether violet light was associated with changes in hypoxia-associated remodeling markers in HSFs. Under hypoxic conditions, violet light exposure further increased OPN5 protein expression compared with hypoxia alone (Figures 2A, B, Supplementary Figure 4). The strongest induction was observed in cells receiving two irradiation sessions.

Violet light exposure was accompanied by changes in several remodeling-associated markers. Relative to hypoxia alone, MMP2 expression was reduced following violet light treatment (Figures 2A, C, Supplementary Figure 4), whereas TIMP2 expression increased (Figures 2A, D, Supplementary Figure 4). COL1A1 protein expression, which was reduced under hypoxia, was partially restored after violet light exposure (Figures 2A, E, Supplementary Figure 4). In addition, α-SMA expression decreased following violet light treatment, with the most pronounced reduction observed in the dual-irradiation group (Figures 2A, F, Supplementary Figure 4).

Together, these observations suggest that violet light further increases OPN5 expression under hypoxic conditions and is associated with partial attenuation of hypoxia-associated changes in remodeling-associated markers.

### Violet light is associated with increased OPN5 expression but limited remodeling-associated molecular changes under normoxic conditions

**FIGURE 3:**
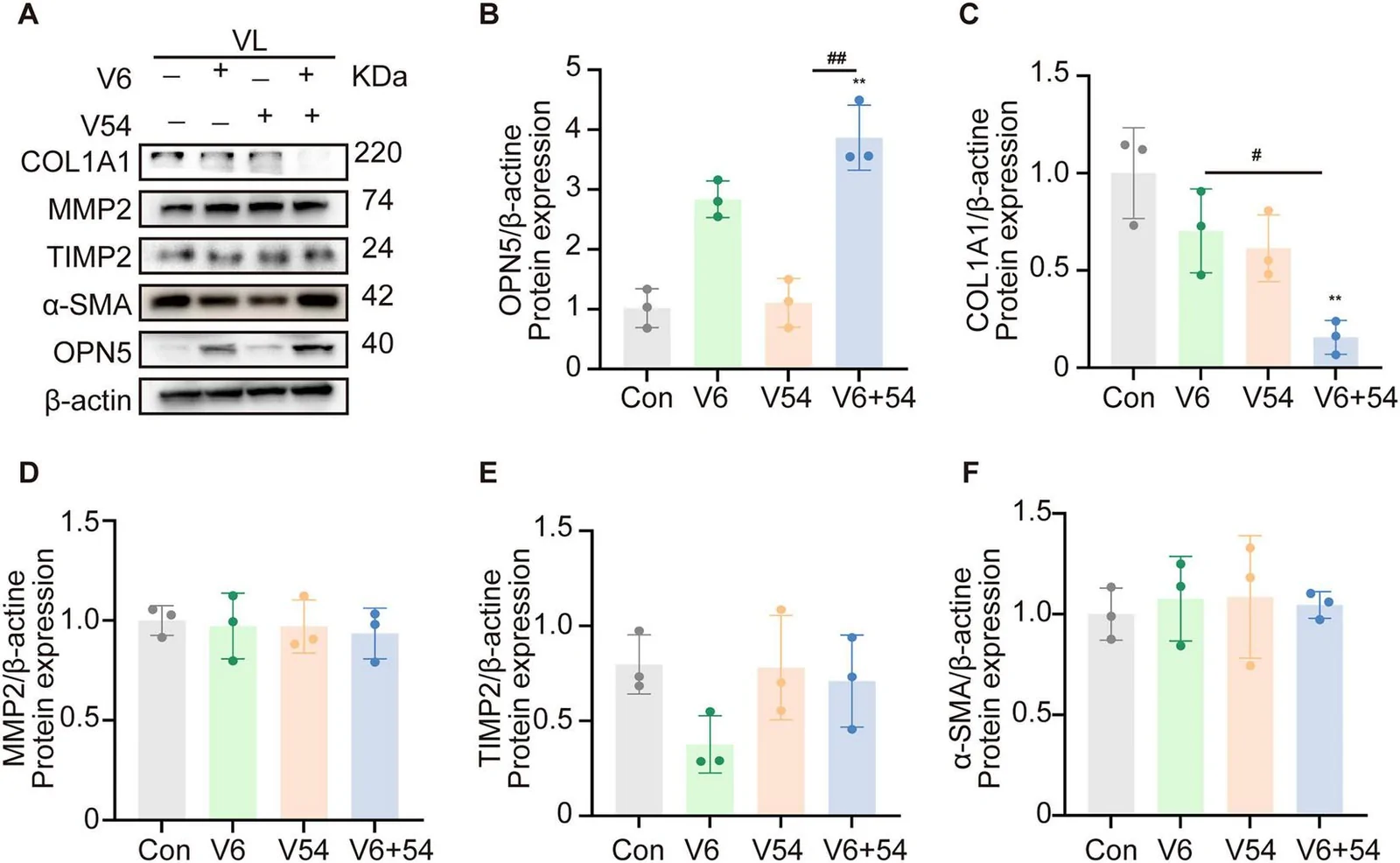
Effects of violet light irradiation under normoxic conditions in HSFs. **(A)** Representative Western blots of COL1A1, MMP2, TIMP2, α-SMA, OPN5, and β-actin in HSFs cultured under normoxic conditions for 72 h with or without violet light irradiation. Violet light (400 μW/cm^2^, 3 h per exposure) was applied at 6 h (V6), at 54 h (V54), or at both time points (V6+V54). **(B–F)** Densitometric quantification of OPN5 **(B)**, COL1A1 **(C)**, MMP2 **(D)**, TIMP2 **(E)**, and α-SMA **(F)** protein expression relative to β-actin (*n* = 3). Violet light increased OPN5 protein expression under normoxia, with the greatest increase observed in the dual-irradiation group, whereas COL1A1 protein expression was significantly decreased only in the V6+V54 group. No significant changes were detected in MMP2, TIMP2, or α-SMA. Data are shown as mean ± SEM. Statistical significance was analyzed by one-way ANOVA followed by Tukey’s *post hoc* multiple-comparison test. */^#^*P* < 0.05, **/^##^*P* < 0.01, ***/^###^*P* < 0.001, and ****/^####^*P* < 0.0001.

To determine whether the effects of violet light depend on hypoxic stress, HSFs were exposed to the same irradiation paradigms under normoxic conditions. Violet light robustly increased OPN5 protein expression, particularly after repeated irradiation (Figures 3A, B), indicating that OPN5 expression remains responsive to violet light under normoxic conditions.

In contrast, changes in remodeling-associated markers were comparatively limited under normoxic conditions.COL1A1 expression showed only a modest reduction after dual irradiation (Figures 3A, C), whereas no significant changes were observed in MMP2, TIMP2, or α-SMA expression (Figures 3A, D–F).

These observations suggest that although violet light consistently increased OPN5 expression, its association with remodeling-associated molecular changes is more evident under hypoxic conditions than normoxic conditions, suggesting that cellular context may influence these responses.

### Increased OPN5 expression is associated with matrix-preserving changes in HSFs

**FIGURE 4:**
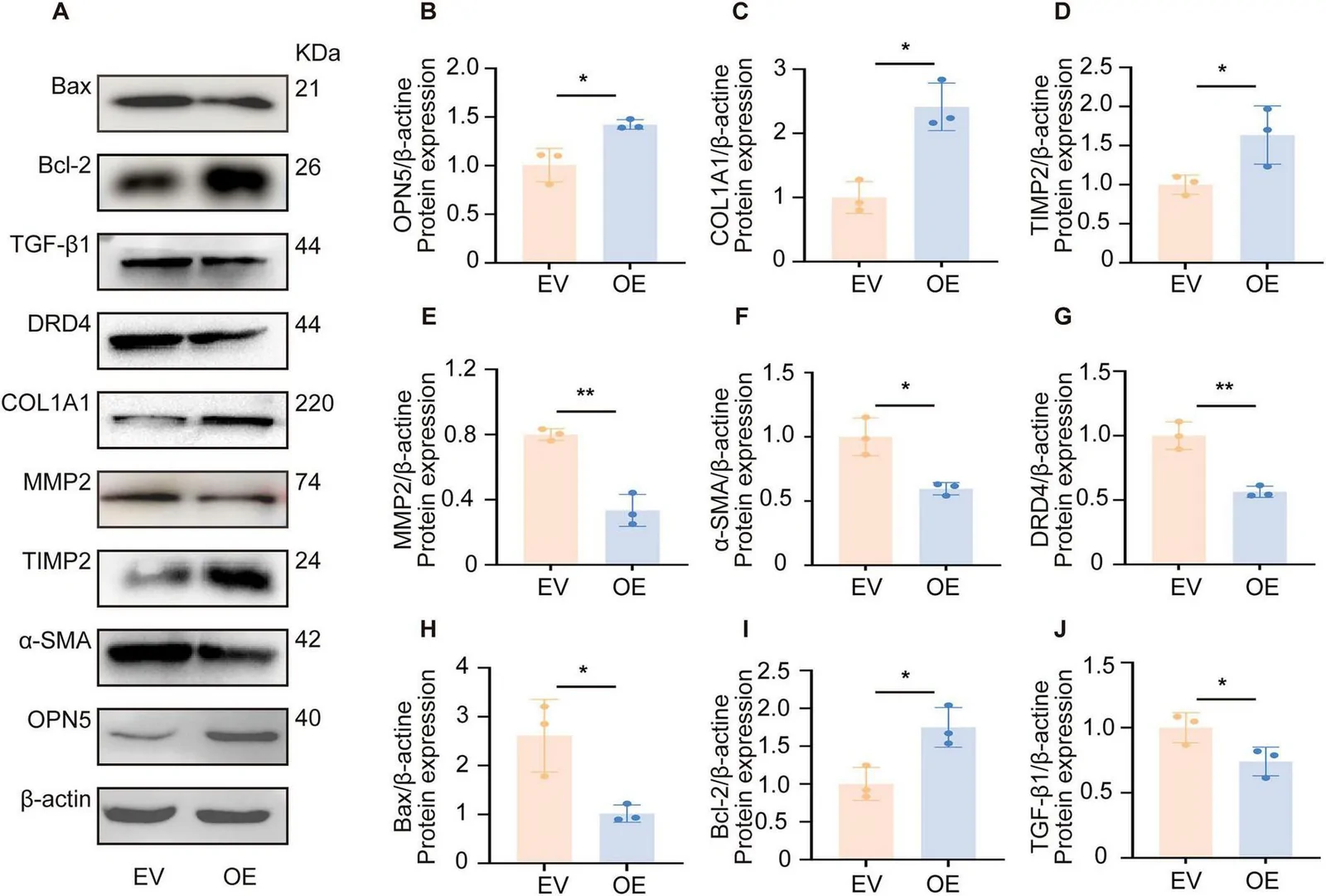
Molecular changes associated with OPN5 overexpression in human scleral fibroblasts. **(A)** Representative Western blots of Bax, Bcl-2, TGF-β1, DRD4, COL1A1, MMP2, TIMP2, α-SMA, OPN5, and β-actin in HSFs transfected with empty vector (EV) or OPN5 overexpression plasmid (OE) for 72 h. **(B–J)** Densitometric quantification of OPN5 **(B)**, COL1A1 **(C)**, TIMP2 **(D)**, MMP2 **(E)**, α-SMA **(F)**, DRD4 **(G)**, Bax **(H)**, Bcl-2 **(I)**, and TGF-β1 **(J)** protein expression relative to β-actin (n = 3). OPN5 overexpression was associated with increased COL1A1, TIMP2, and Bcl-2, but reduced MMP2, α-SMA, DRD4, Bax, and TGF-β1. Data are shown as mean ± SEM. Statistical analysis was performed using Student’s *t*-test. *P < 0.05, **P < 0.01.

To further examine the relationship between OPN5 and extracellular matrix-associated pathways, OPN5-overexpressing HSFs were established and validated by increased OPN5 expression at both the transcript and protein levels (Figures 4A, B, Supplementary Figure 5).

OPN5 overexpression was associated with increased COL1A1 and TIMP2 expression at both the mRNA and protein levels (Figures 4A, C, D, Supplementary Figure 5C). In contrast, MMP2 and α-SMA protein expression were reduced in OPN5-overexpressing cells (Figures 4A, E, F), consistent with a molecular profile suggestive of reduced fibroblast activation.

Additional changes were also observed in stress-associated markers. DRD4 and Bax protein expression decreased following OPN5 overexpression, whereas Bcl-2 expression increased (Figures 4A, G–I). TGF-β1 showed discordant transcript and protein changes, with increased mRNA expression but reduced protein abundance (Figures 4A, J, Supplementary Figure 5). Such discrepancies may reflect post-transcriptional regulation, differences in translation efficiency, altered protein stability or degradation, or feedback mechanisms that influence TGF-β1 protein abundance (Fraser et al., 2008; Wu et al., 2023) independently of transcript levels. Additional mechanistic studies will be required to clarify the basis of these observations. Overall, increased OPN5 expression was associated with molecular signatures consistent with enhanced extracellular matrix homeostasis and reduced fibroblast activation.

### Reduction of endogenous OPN5 expression is associated with molecular changes consistent with impaired extracellular matrix homeostasis

**FIGURE 5:**
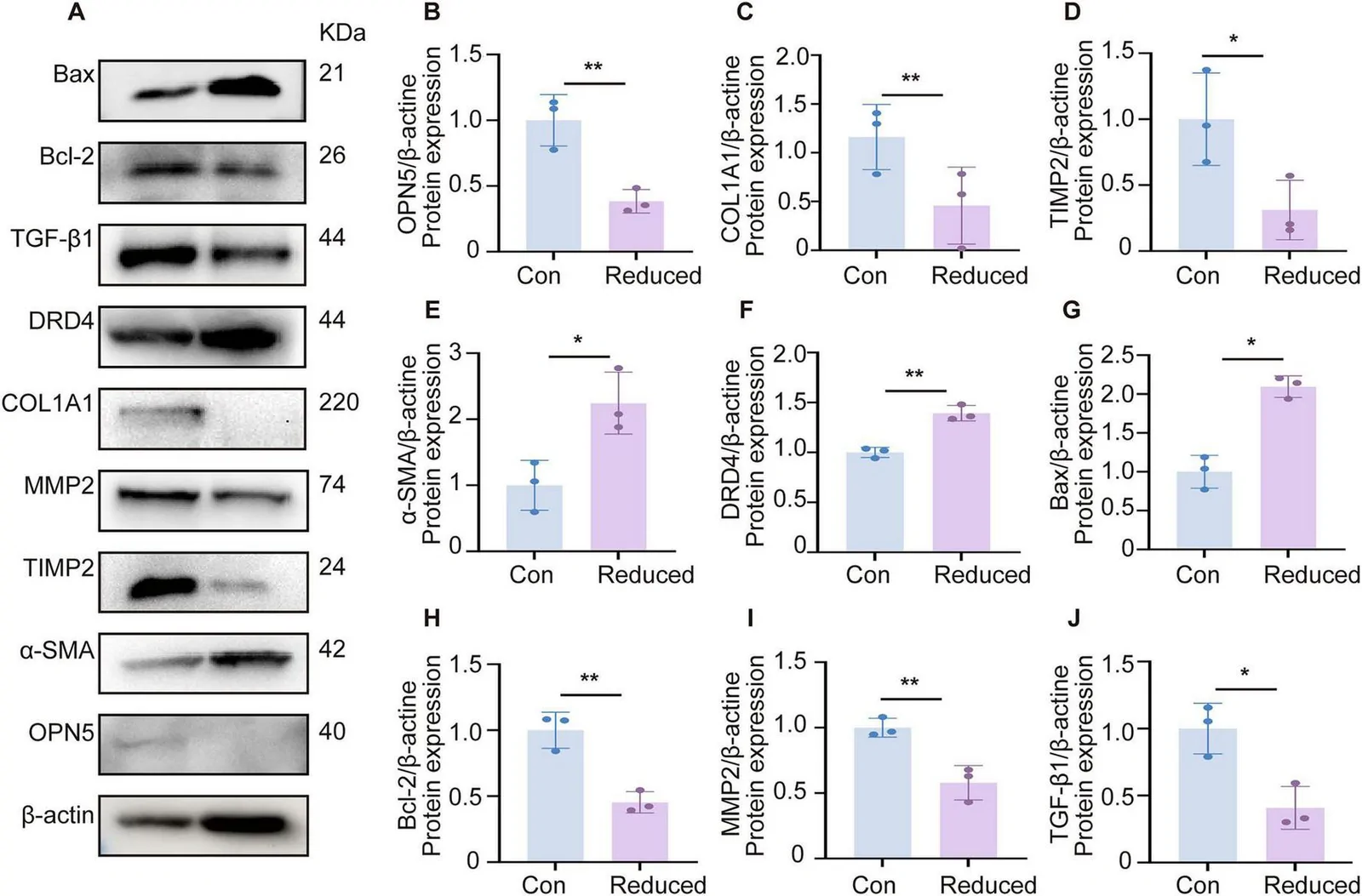
Molecular changes associated with CRISPR-mediated OPN5 reduction in human scleral fibroblasts. **(A)** Representative Western blots of Bax, Bcl-2, TGF-β1, DRD4, COL1A1, MMP2, TIMP2, α-SMA, OPN5, and β-actin in control (Con) and OPN5-reduced HSFs. **(B–J)** Densitometric quantification of OPN5 **(B)**, COL1A1 **(C)**, TIMP2 **(D)**, α-SMA **(E)**, DRD4 **(F)**, Bax **(G)**, Bcl-2 **(H)**, MMP2 **(I)**, and TGF-β1 **(J)** protein expression relative to β-actin (*n* = 3). OPN5-reduced was associated with reduced COL1A1, TIMP2, Bcl-2, and TGF-β1 protein expression together with increased α-SMA, DRD4, and Bax levels. MMP2 showed inconsistent transcript and protein changes across analyses. Data are shown as mean ± SEM. Statistical significance was analyzed using Student’s *t*-test. *P < 0.05, **P < 0.01. Reduced, CRISPR-mediated OPN5 reduction.

To complement the overexpression experiments, HSFs with CRISPR-mediated reduction of OPN5 expression were established. Although stable monoclonal knockout cells were not obtained, OPN5 protein expression was markedly reduced compared with control cells (Figures 5A, B). Western blot analysis demonstrated an approximately ∼71% reduction in OPN5 protein expression compared with control cells (Supplementary Figure 2).

Reduction of endogenous OPN5 expression was accompanied by decreased COL1A1 and TIMP2 expression (Figures 5A, C, D, Supplementary Figure 6B). In contrast, α-SMA expression increased at both the transcript and protein levels (Figures 5A, E, Supplementary Figure 6), consistent with molecular features of increased fibroblast activation.

Changes were also observed in additional stress-associated markers. DRD4 and Bax protein expression increased following reduction of OPN5 expression, whereas Bcl-2 expression decreased (Figures 5A, F–H). MMP2 showed discordant transcript and protein changes, with increased mRNA expression but reduced protein abundance (Figures 5A, I, Supplementary Figure 6). Similar transcript–protein discrepancies have been reported for MMP2 in other biological systems (Lichtinghagen et al., 2002), suggesting that MMP2 is regulated at multiple levels beyond transcription. These findings raise the possibility that reduction of OPN5 expression may influence post-transcriptional or post-translational regulation of MMP2 rather than transcription alone, although the underlying mechanisms remain to be determined. TGF-β1 expression decreased at both transcript and protein levels (Figures 5A, J, Supplementary Figure 6).

Taken together, these findings suggest that endogenous OPN5 expression is associated with molecular features consistent with extracellular matrix homeostasis in HSFs.

## Discussion

Scleral extracellular matrix remodeling is a central structural feature of myopia progression and is increasingly linked to local hypoxic stress within the posterior eye. In the present study, hypoxia induced coordinated changes in human scleral fibroblasts characterized by reduced COL1A1 and TIMP2 expression together with increased MMP2 and α-SMA expression. These observations are consistent with previous studies reporting that hypoxia is associated with increased matrix turnover and fibroblast activation in the sclera (Peng et al., 2026; Wang et al., 2025; Wu et al., 2018). Rather than representing isolated molecular alterations, the concurrent changes across multiple remodeling-associated markers suggest a coordinated remodeling response in HSFs.

One notable finding of the present study was the progressive increase in OPN5 expression under hypoxic conditions. Although OPN5 has primarily been characterized as a violet light-sensitive opsin involved in retinal and circadian physiology (Buhr et al., 2015; Jiang et al., 2021), comparatively little is known about its role in scleral fibroblasts. The parallel induction of OPN5 and remodeling-associated markers suggests that OPN5 may participate in hypoxia-responsive signaling pathways. However, the present data do not establish whether OPN5 acts as a driver of, or a compensatory response to, hypoxia-induced remodeling. Because hypoxia-induced scleral remodeling is generally considered to be mediated by HIF-1α signaling (Wu et al., 2018; Zhao et al., 2020). These findings raise the possibility that OPN5 induction may be associated with HIF-1α signaling. Nevertheless, the present study was not designed to determine whether OPN5 is a direct transcriptional target of HIF-1α. Future mechanistic studies incorporating promoter analysis together with pharmacological or genetic inhibition of HIF-1α will be required to clarify the regulatory relationship between HIF-1α and OPN5.

Consistent with the observations in chick eyes reported by Kato et al. (2019), our findings extend the potential involvement of OPN5 to hypoxia-associated molecular responses in human scleral fibroblasts. Whereas the previous study focused primarily on retinal development during embryogenesis, the present work examined extracellular matrix-related molecular changes in adult scleral fibroblasts under hypoxic conditions. Together, these observations suggest that OPN5 may participate in distinct biological processes depending on developmental stage and tissue context. These studies support the concept that OPN5 is responsive to violet light, although the downstream biological contexts differ substantially.

Gain- and loss-of-function experiments consistently associated higher OPN5 expression with increased COL1A1 and TIMP2 expression and reduced α-SMA expression, supporting an association between OPN5 expression and molecular signatures consistent with extracellular matrix homeostasis. In contrast, the effects on MMP2 were less consistent, particularly in OPN5-reduced cells, where transcript and protein expression were discordant. Together, these findings suggest that OPN5 may influence extracellular matrix homeostasis through broader stress-responsive signaling networks rather than through direct regulation of individual remodeling-associated molecules.

Violet light further enhanced OPN5 expression and was associated with partial attenuation of hypoxia-associated remodeling changes, whereas comparatively limited downstream molecular responses were observed under normoxic conditions. These observations suggest that OPN5-associated signaling is context dependent and may become functionally more relevant under hypoxic stress. Although violet light consistently increased OPN5 expression and gain- and loss-of-function experiments demonstrated consistent associations between OPN5 expression and extracellular matrix-related markers, these findings do not establish that violet light exerts its remodeling-associated effects exclusively through OPN5. Defining the causal contribution of OPN5 will require complementary approaches, including rescue experiments, pharmacological or genetic inhibition, genetic complementation, and evaluation of whether violet light responses are abolished in OPN5-reduced cells. Such studies will help clarify the relationship among violet light exposure, OPN5 activation, and extracellular matrix remodeling, and determine the extent to which OPN5 directly mediates light-associated remodeling responses in scleral fibroblasts.

During hypoxic exposure, COL1A1 mRNA exhibited a transient increase at 24 h, whereas protein expression progressively declined. This early transcriptional response may reflect an initial compensatory adaptation to hypoxic stress, whereas the subsequent reduction in protein abundance may involve post-transcriptional regulation. Previous studies have identified the miR-29 family as a key regulator of collagen synthesis (van Rooij et al., 2008), and HIF-1α signaling has been reported to modulate miR-29 expression under hypoxic conditions in several tissues (Fang et al., 2013; Lee et al., 2020; Schofield and Ratcliffe, 2004). Whether a similar mechanism contributes to COL1A1 regulation in human scleral fibroblasts remains to be determined. In addition, discordant transcript and protein expression patterns were also observed for TGF-β1 in OPN5-overexpressing cells and MMP2 in OPN5-reduced cells. Due to a lack of mechanistic data, the functional significance of their discordant expression is unclear. It is speculated that these discrepancies may reflect post-transcriptional regulation, translational control, protein turnover, or feedback regulatory mechanisms, indicating that transcript abundance may not necessarily predict protein expression under these conditions. Altered Bax/Bcl-2 and DRD4 expressions further suggest that OPN5 may be associated with broader stress-responsive pathways beyond extracellular matrix remodeling. Future studies integrating functional analyses of apoptosis, dopamine signaling, and protein regulatory mechanisms will be necessary to clarify the biological significance of these observations.

Transcriptomic analysis further suggested potential associations between OPN5 expression and extracellular matrix regulation, lipid metabolism (Supplementary Figure 7F), Wnt signaling, and cytokine-responsive pathways (Supplementary Figure 7T). Because these analyses were exploratory and lacked functional validation, they should primarily be regarded as hypothesis-generating. Interestingly, several of the pathways identified in our RNA-seq analysis, including extracellular matrix remodeling, Wnt signaling, and inflammatory responses, have been reported to be extensively regulated by epigenetic mechanisms during myopia progression. Recent studies have highlighted important roles for RNA epigenetic modifications, particularly m6A and m5C methylation, in regulating hypoxia-associated scleral remodeling. For example, the m6A demethylase ALKBH5 promotes myopia progression through ERK1/2-dependent signaling (Zhu J. et al., 2025), whereas the m5C demethylase TET1 regulates hypoxia-associated extracellular matrix remodeling in human scleral fibroblasts (Jia et al., 2026). Given that the present study identified OPN5 as a hypoxia-responsive factor associated with extracellular matrix homeostasis, these findings raise the possibility that epigenetic regulation may constitute an additional regulatory layer linking hypoxic stress to OPN5-associated extracellular matrix remodeling. Although these possibilities remain speculative, future studies integrating epigenetic profiling with functional analysis of OPN5 signaling may provide important mechanistic insight into scleral remodeling during myopia progression.

Several limitations should be acknowledged. First, the present findings were obtained exclusively from cultured human scleral fibroblasts, and validation in animal models together with human myopic scleral tissues will be essential to establish their physiological relevance. Second, the study focused primarily on molecular marker expression rather than functional outcomes such as extracellular matrix biomechanics, collagen organization, or axial elongation, which should be evaluated in future investigations. Third, the CRISPR/Cas9-edited cells represented a heterogeneous bulk population rather than monoclonal knockout cell lines, introducing the possibility of residual OPN5 expression, variable editing efficiency, and off-target effects. Confirmation using transient siRNA-mediated silencing or stable monoclonal knockout models would strengthen these observations. Finally, additional mechanistic studies incorporating rescue experiments, pathway-specific inhibition, and HIF-1α validation will be important for determining whether OPN5 directly mediates hypoxia- and violet light-associated remodeling responses.

In summary, hypoxia induced coordinated remodeling-associated molecular changes together with OPN5 upregulation in human scleral fibroblasts. Increased OPN5 expression was consistently associated with molecular signatures indicative of enhanced extracellular matrix homeostasis, whereas violet light further enhanced OPN5 expression and was associated with partial attenuation of hypoxia-associated remodeling changes. Collectively, these findings identify OPN5 as a hypoxia-responsive factor in scleral fibroblasts and support a context-dependent association between OPN5 signaling and extracellular matrix homeostasis under conditions relevant to myopia-associated scleral remodeling.

## Data Availability

All raw data generated in this study are included in the main text and Supplementary Materials. The transcriptomic data generated in this study have been deposited in the Genome Sequence Archive in National Genomics Data Center, China National Center for Bioinformation / Beijing Institute of Genomics, Chinese Academy of Sciences (GSA-Human: HRA020188) that are publicly accessible at https://ngdc.cncb.ac.cn/gsa-human. Further information regarding the data is available from the corresponding author upon reasonable request.
