# Supplementary material for: Hypoxia-induced OPN5 expression is associated with matrix-preserving responses in human scleral fibroblasts
Source: Front Neurosci. 2026 Aug 10;20:1896819. doi: 10.3389/fnins.2026.1896819 (PMC13501907; doi:10.3389/fnins.2026.1896819)
Supplement: Supplementary file 1 [file Data_Sheet_1.docx]

**Supporting Information for**

***Hypoxia-Induced OPN5 Expression Is Associated with Matrix-Preserving Responses in Human Scleral Fibroblasts***

**This PDF file includes:**

Figures and Legends S1 to S7

Tables S1 to S2

**Fig. S1. Temperature monitoring and Reactive Oxygen Species (ROS) during violet light** **irradiation.**

A. Temperature was measured at the culture plate surface during violet light irradiation under the experimental conditions used in this study. No appreciable increase in temperature was observed throughout the irradiation period, indicating that violet light exposure did not produce detectable thermal effects under the conditions employed. **B.** Representative bright-field and DCFH-DA fluorescence images of HSFs cultured under normoxic conditions for 72 h with or without violet light irradiation. Violet light (400 μW/cm², 3 h per irradiation) was applied at 6 h (V6), 54 h (V54), or both time points (V6+V54). Quantification of DCFH-DA fluorescence intensity revealed no significant differences between violet light-treated groups and the normoxic control. **C.** Representative bright-field and DCFH-DA fluorescence images of HSFs cultured under hypoxic conditions (2% O₂, 72 h) with or without violet light irradiation using the same irradiation protocol. Quantitative analysis likewise showed no significant differences in intracellular ROS levels between violet light-treated groups and the hypoxic control. Fluorescence intensity was quantified using ImageJ from at least three randomly selected fields per group in three independent experiments.

**Fig. S2.** **CRISPR-mediated OPN5 reduction in human scleral fibroblasts.**

**(A-E).** Representative Western blot results from five independent repeated experiments are shown, with β-actin used as the loading control. Densitometric quantification of OPN5 protein levels normalized to β-actin demonstrated reduced OPN5 expression in the CRISPR-treated groups compared with controls, with knockdown efficiencies of 59.7%, 81.7%, 55.5%, 68.8%, and 89.6% across the five repeats. **F.** The summary plot shows the mean OPN5 reduction efficiency of 71.6%. Data are presented as mean ± SD.

**Fig. S3. Hypoxia induces OPN5 and remodeling-associated transcriptional changes in human scleral fibroblasts.**

(A–E) Human scleral fibroblasts (HSFs) were cultured under hypoxic conditions (2% O₂) for 0, 12, 24, 48, or 72 h. Relative mRNA expression levels of OPN5 (A), COL1A1 (B), MMP2 (C), TIMP2 (D), and α-SMA (E) were analyzed by quantitative real-time PCR and normalized to β-actin using the 2^−ΔΔCt method (n = 3). Hypoxia progressively increased OPN5, MMP2, and α-SMA expression, whereas TIMP2 expression decreased over time. COL1A1 expression showed a transient increase at 24 h followed by a decline at later time points. Data are presented as mean ± SEM. Statistical analysis was performed using one-way ANOVA followed by Tukey’s multiple-comparison test. */# P < 0.05, **/## P < 0.01, ***/### P < 0.001, ****/#### P < 0.0001.

**Fig. S4. Violet light modulates remodeling-associated gene expression under hypoxic conditions.**

(A–E) Human scleral fibroblasts (HSFs) were cultured under hypoxia (2% O₂) for 72 h and exposed to violet light irradiation (400 μW/cm², 3 h per session) at 6 h (V6), 54 h (V54), or both time points (V6+V54). Relative mRNA expression levels of OPN5 (A), MMP2 (B), TIMP2 (C), COL1A1 (D), and α-SMA (E) were measured by quantitative real-time PCR and normalized to β-actin using the 2^−ΔΔCt method (n = 3). Violet light increased OPN5, TIMP2, and COL1A1 expression and reduced MMP2 and α-SMA expression, with the most prominent effects observed in the dual-irradiation group. Data are presented as mean ± SEM. Statistical analysis was performed using one-way ANOVA followed by Tukey’s multiple-comparison test. */# P < 0.05, **/## P < 0.01, ***/### P < 0.001, ****/#### P < 0.0001.

**Fig. S5. OPN5 overexpression alters extracellular matrix-associated gene expression in human scleral fibroblasts.**

(A–D) Human scleral fibroblasts (HSFs) were transfected with an OPN5 overexpression plasmid (OE) or empty vector control (EV). After 72 h, relative mRNA expression levels of OPN5 (A), COL1A1 (B), TIMP2 (C), and TGF-β1 (D) were analyzed by quantitative real-time PCR and normalized to β-actin using the 2^−ΔΔCt method (n = 3). OPN5 overexpression increased the expression of OPN5, COL1A1, TIMP2, and TGF-β1. Data are presented as mean ± SEM. Statistical analysis was performed using an unpaired two-tailed Student’s t-test. *** P < 0.001, **** P < 0.0001.

**Fig. S6. Reduction of endogenous OPN5 alters remodeling-associated gene expression in human scleral fibroblasts.**

(A–E) Human scleral fibroblasts (HSFs) transfected with CRISPR/Cas9 constructs targeting OPN5 (KD) or control cells (Con) were analyzed by quantitative real-time PCR. Relative mRNA expression levels of COL1A1 (A), TIMP2 (B), α-SMA (C), MMP2 (D), and TGF-β1 (E) were normalized to β-actin using the 2^−ΔΔCt method (n = 3). OPN5 reduction decreased COL1A1, TIMP2, and TGF-β1 expression, whereas α-SMA and MMP2 expression increased. Data are presented as mean ± SEM. Statistical analysis was performed using an unpaired two-tailed Student’s t-test. * P < 0.05, ** P < 0.01.

**Fig. S7. Transcriptomic profiling of OPN5-overexpressing HSFs identifies candidate pathways associated with extracellular matrix regulation and stress-responsive signaling.**

(A, G, N) Volcano plots showing differentially expressed genes in OPN5-overexpressing human scleral fibroblasts (OE) compared with empty vector controls (EV) identified by bulk RNA sequencing (n = 3 per group). Upregulated genes are shown in red, downregulated genes in blue, and non-significant genes in gray. (B–F) Relative expression levels (FPKM) of selected genes associated with lipid metabolism and nuclear receptor signaling, including RXRA, NR1H3, FABP3, PLIN4, and CPT1C. (H–M) Relative expression levels (FPKM) of selected genes associated with Wnt-related signaling, including CTNNBIP1, NOTUM, PRICKLE3, TLE2, LZTS2, and APC2. (O–T) Relative expression levels (FPKM) of selected cytokine- and stress-associated genes, including SOCS1, CISH, IL11RA, SERPINF1, IL6, and IL7R. Data are presented as mean ± SEM. Statistical significance is indicated in the corresponding panels.

**Supplementary Table S1.**

| Primary antibody | SOURCE | Catalog No. | Dilution ratio |
| --- | --- | --- | --- |
| OPN5 | ABclonal, Wuhan, China | A15971 | 1:750 |
| MMP2 | Proteintech, Wuhan, China | 10373-2-AP | 1:1000 |
| COL1A1 | Abcam, USA | ab255809 | 1:1000 |
| TIMP2 | ABclonal, Wuhan, China | A20766 | 1:750 |
| β-actin | Abcam, USA | ab241153 | 1:1000 |
| DRD4 | Proteintech, Wuhan, China | 28094-1-AP | 1:1000 |
| α-SMA | Abcam, USA | ab124936 | 1:50000 |
| TGF-β1 | Abways, Shanghai, China | CY6608 | 1:1000 |
| Bax | Abcam, USA | ab32503 | 1:5000 |
| Bcl-2 | Abcam, USA | ab32124 | 1:1000 |

**Supplementary Table S2.**

| Primer | 5' to 3' |
| --- | --- |
| OPN5-Human-F | GCCGTTCACCATCATCTCTT |
| OPN5-Human-R | TAAGGCTTCCACAGCCAAAG |
| COL1A1-Human-F | TGCGATGACGTGATCTGTGA |
| COL1A1-Human-R | TTTCTTGGTCGGTGGGTGA |
| β-actin-Human-F | GTCCACCGCAAATGCTTCTA |
| β-actin-Human-R | TGCTGTCACCTTCACCGTTC |
| MMP2-Human-F | CTCATCGCAGATGCCTGGAA |
| MMP2-Human-R | TTCAGGTAATAGGCACCCTTGAAGA |
| TIMP2-Human-F | TCATTGCAGGAAAGGCCGAG |
| TIMP2-Human-F | GGCTCTTCTTCTGGGTGGTG |
| TGF-β1-Human-F | CAATTCCTGGCGATACCTCAG |
| TGF-β1-Human-R | GCACAACTCCGGTGACATCAA |
| α-SMA-Human-F | TGGCTATTCCTTCGTTACTACTGCT |
| α-SMA-Human-R | CATCAGGCAACTCGTAACTCTTCTC |
